# Mechanical, Thermal, and Fire Properties of Composite Materials Based on Gypsum and PCM

**DOI:** 10.3390/ma15031253

**Published:** 2022-02-08

**Authors:** Kateřina Stejskalová, David Bujdoš, Lukáš Procházka, Bedřich Smetana, Simona Zlá, Jiří Teslík

**Affiliations:** 1 Faculty of Civil Engineering, VSB—Technical University of Ostrava, 70800 Ostrava, Czech Republic; david.bujdos@vsb.cz (D.B.); lukas.prochazka@vsb.cz (L.P.); jiri.teslik@vsb.cz (J.T.); 2 Faculty of Materials Science and Technology, VSB—Technical University of Ostrava, 70800 Ostrava, Czech Republic; bedrich.smetana@vsb.cz (B.S.); simona.zla@vsb.cz (S.Z.)

**Keywords:** fire properties, gypsum, mechanical properties, phase change material, thermal properties

## Abstract

One of the solutions for overheating the interior in the summer without increasing energy consumption is the integration of phase change material (PCM) into interior plasters. However, adding PCM to plasters deteriorates their properties and thus their usability. The aim of this paper is to determine how the microencapsulated PCM affects the mechanical, thermal, and fire properties of plasters and how much PCM can be added to the plaster. Two sets of samples were prepared: in set S, part of the aggregate was replaced by PCM; and in set R, only PCM was added. The bulk density, flexural strength, compressive strength, tensile strength perpendicular to the surface, thermal conductivity coefficient, specific heat capacity, melting, and solidification temperatures and enthalpy were measured. A single-flame source fire test and a gross heat of combustion fire test were performed to determine the reaction to the fire class. The results show that with an increasing proportion of PCM, the strength of the samples of set R decreased more significantly than it did with the samples of set S. It was found that only up to about 10% PCM could be added to set R, while up to 30% PCM could be added to set S.

## 1. Introduction

Overheating of rooms in the summer months is a common problem, especially when using lightweight structures. Recently, more attention has been paid to this topic; it is necessary to find a long-term effective solution to this problem. With the right design, it is possible to reduce interior overheating in the summer months to ensure thermal comfort for the occupants. The simplest solution is the use of air conditioning, but its energy consumption during operation has a negative impact on the environment. One solution without the need for power consumption for operation is the use of phase change material (PCM) [1]. PCM is able to store/release latent heat at normal indoor air temperatures [2,3]. When the temperature in the interior reaches the melting temperature of the material, a phase change (melting) occurs in the material, during which energy is stored in the form of latent heat [4]. The heat storage in the PCM reduces the indoor temperature. Conversely, when the indoor air temperature drops below the solidification temperature, the material solidifies back and releases heat into the interior. This increases the temperature in the interior and saves energy required for heating. There are several ways to integrate PCM into a building’s structure [5,6]; this paper focuses on the integration of PCM into interior plasters [7].

Although the integration of PCM into plaster mixtures will increase the heat storage capacity, it might worsen the mechanical properties, thermal conductivity [8,9,10,11,12], and fire resistance of the plaster [13,14,15,16]. The aim of this research is to get as much PCM as possible into the plaster mixture, but at the same time meet the requirements for the usability, strength, and fire resistance of the plaster [17,18]. There are many materials used as PCM [4]; microencapsulated PCM was used in this research. Zhuk [19] found in a lifecycle analysis that gypsum plaster with microencapsulated PCM could withstand over 10,000 temperature load cycles without losing its properties, which corresponds to more than 30 years of minimal service life according to RAL-GZ 896 [20]. PCM has the function of an aggregate in the mixture. However, due to its fine structure, it cannot completely replace the aggregate, so PCM forms only a part of the aggregate in the plaster mixture. The first option is to create completely new mixtures, in which a part of the aggregate is replaced by PCM [21,22,23], and the second option is to add PCM to the commercial dry plaster mixture [24,25,26]. In the second option, it is not possible to get too much PCM into the plaster, because its addition increases the amount of aggregate in the mixture, but it is very easy to prepare such a plaster on the construction site. In this paper, both possibilities of creating a plaster mixture were tested and compared. Several research groups have investigated the mechanical and thermal properties of PCM plasters [27]. Bajare et al. [8] found that adding 10% microencapsulated PCM to a cement–lime plaster reduced its flexural strength by 40% and its compressive strength by 60%. Pavlík et al. [9] added 8%, 16%, and 24% PCM to a commercial plaster mixture and investigated the basic physical, mechanical, and thermal properties. The additional phase change enthalpy was up to 13 J/g, but the mechanical parameters decreased by up to 40%, which means that the plasters did not meet the requirements of the standard [17]. Fořt et al. [24] presented similar research with 4%, 8%, and 12% PCM in a commercial plaster mixture. Based on previous research, Fořt et al. [28] evaluated the designed plasters by computational analysis. Klimeš et al. [29] developed an optimization model of a thermally activated wall system with a gypsum plaster containing a microencapsulated PCM. Pavlík et al. [21] presented the results of a DSC analysis (melting and crystallization peak temperature and heat) of lime–pozzolan plaster with 5%, 10%, and 15% of PCM. DSC analysis of lime–pozzolan plaster with 5% was also performed by Pavlíková et al. [30]. Kusama et al. [23] performed a laboratory and residential-scale test of the PCM gypsum plaster. The PCM plaster offered a high solar radiation effective utilization rate (82%). Zhou et al. [31] numerically evaluated a PCM–gypsum composite in a passive solar building in Beijing with an enthalpy model. The PCM–gypsum composite effectively shaved the indoor temperature swing by 46%. Theodoridou et al. [32] added 5% PCM into plasters with hydrated lime or hydraulic lime and investigated their thermal, physical, and mechanical properties. The plasters with hydrated lime did not meet the requirements of the standard [17]. Kheradmand et al. [33] compared a modified cement plaster containing 18.34% PCM to a reference plaster. Compared to the reference plaster without PCM, a 20% reduction in energy consumption for heating/cooling was found. Kusama et al. [34] evaluated the basic thermal performance and energy-saving effects of a system containing a PCM plaster. The thermal energy-saving effects in Hokkaido, Japan were approximately 52%. Carbonaro et al. [35] added 14% PCM with melting temperatures of 26 °C (V1) and 23 °C (V2) into a plaster based on lime and gypsum. The thermal conductivities at 6, 21, and 36 °C was measured: for the plaster with PCM V1, there was a reduction of 68%; and for PCM V2 there was a reduction of 47%. To improve the thermal conductivity of a plaster, it is possible to add natural or expanded graphite [22] or aluminum [36] to the mixture. To increase the heat storage/release, plasters can be impregnated with PCM [37,38,39]. Several studies have also focused on the fire properties of building materials containing PCM. Some have researched plasterboards [14,40], and others have researched plasters [15,41]. Using new types of PCM [42,43], improved coatings [41], or bio-PCM [40], it is possible to increase the fire resistance of a structure. To determine the usability of a plaster in the interior, the reaction to fire class is also important, which this paper will extend beyond the mentioned research.

In this research, the PCM Micronal DS 5008 X (manufactured by BASF SE, Ludwigshafen, Germany) was selected. It is a fine powder consisting of paraffin wax encapsulated in polymeric spherical microcapsules [44]. Gypsum, which is commonly used for interior plasters, was chosen as the binder [45]. Two sets of samples with an increasing proportion of PCM of approximately 5–10% were prepared. In the first set, part of the aggregate was replaced by PCM, and in the second set, PCM was added to the commercial gypsum plaster mixture. The mechanical, thermal, and fire properties of these samples were determined. The aim of this research was to determine the dependence of individual properties on the amount of PCM in the plaster mixture so that it was possible to better predict how a given amount of PCM in a mixture will affect its properties [46]. From the mechanical properties, the flexural strength *P_F_* (MPa), the compressive strength *R_C_* (MPa), and the tensile strength perpendicular to the surface *R_u_* (MPa) were measured. From the thermal properties, the thermal conductivity coefficient and the specific heat capacity were measured. A DSC (differential scanning calorimetry) analysis determined the melting and solidification temperatures and latent heat of the developed composite material. From the fire properties, the reaction to fire class of the composite material was determined. Gypsum and aggregates are non-flammable materials of class A1 [47], but PCM is made of paraffin wax and polymer, which are highly flammable. Therefore, a single-flame source fire test and a gross heat of combustion fire test were used to determine how PCM degrades the fire resistance of the developed composite material and in which reaction to fire class according to [47] the composite material would be classified.

This paper first describes the materials used and the process for producing individual gypsum plaster samples with different proportions of PCM. The next chapter describes methods for measuring individual mechanical, thermal, and fire properties. This is followed by a description and analysis of the results obtained by that measurement. Finally, the results of both sets are compared.

## 2. Materials and Methods

### 2.1. Materials Used

For the production of the composite, gypsum, fine quartz sand (0.09–1.25 mm), commercial gypsum plaster mixture, and PCM were selected. PCM Micronal DS 5008 X was used in all test samples. Two sets of test samples with an increasing proportion of PCM were prepared. For the first set, gypsum (CaSO_4_·1/2 H_2_O) class G5 [48] was used as a binder and sand and PCM as aggregate. As the proportion of PCM in the mixture increased, the amount of sand decreased. This set was marked as S. The second set was made of commercial gypsum plaster mixture (Rigips Rimano UNI) and PCM. Rigips Rimano UNI plaster mixture contains gypsum with additives that improve application and adhesion, lime hydrate and lightweight expanded perlite [49]. This set was marked as R. The addition of PCM as aggregate to the commercial mixture increases the amount of aggregate in the composite, and therefore it was not possible to add as much PCM to this set as to set S. Samples S0 and R0 were reference and did not contain PCM.

### 2.2. Production Process

A total of eight test samples were produced in set S and five samples in set R. First, the dry components of the mixture were weighed according to Table 1 and Table 2. The amount of PCM and sand was determined so that in each sample the ratio of aggregate volume to gypsum volume was the same as it was in reference samples S0 and R0. Mixtures of dry components and a sample containing only PCM were used for differential scanning calorimetry and gross heat of combustion measurement. To measure the other properties, water was gradually added in the amounts shown in Table 1 and Table 2. The mixture was mixed for about one minute.

**Figure 1:**
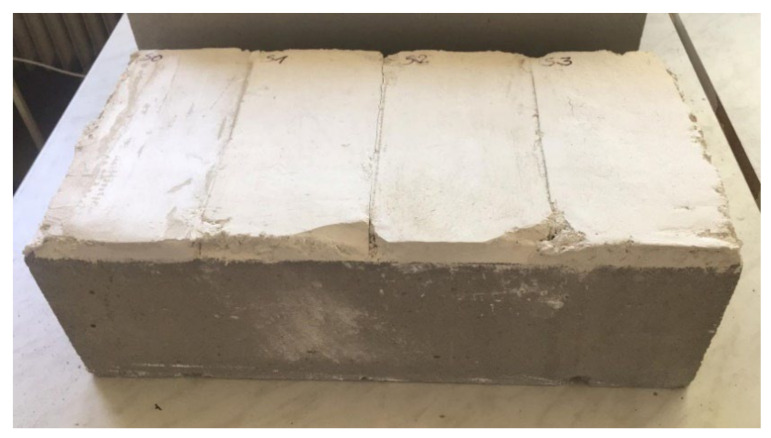
Samples to measure the tensile strength perpendicular to the surface.

To measure the tensile strength perpendicular to the surface, a layer of each plaster mixture was applied to the aerated concrete blocks (Figure 1). The surface of the aerated concrete blocks was penetrated and a layer of each plaster mixture approximately 20 mm thick was applied. These samples were left in the test environment (temperature 23 ± 2 °C and relative air humidity 50 ± 5%) for 28 days and then tested [50].

**Figure 2:**
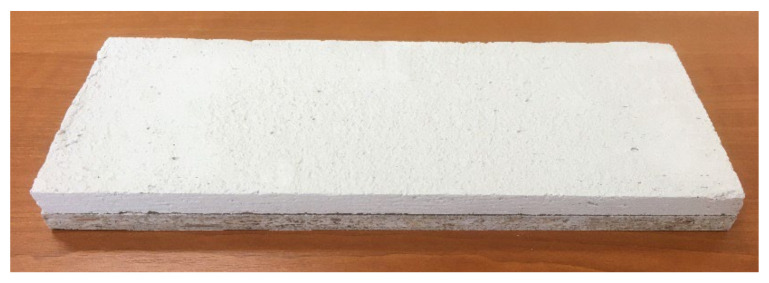
Sample for the single-flame source fire test.

For the single-flame source fire test, samples measuring 250 × 90 × 15 mm were made [51]. First, a base measuring 250 × 90 mm and a form were made of 8 mm thick chipboard. These forms were filled with individual plaster mixtures, and the surface of the samples was smoothed with a trowel. These samples were stored in the test environment (temperature 23 ± 2 °C and relative air humidity 50 ± 5%) for 28 days [50], then removed (Figure 2) and tested.

**Figure 3:**
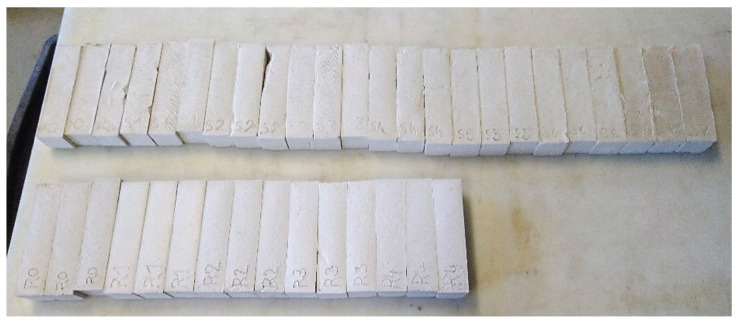
Samples from plastic molds.

For other tests, samples with dimensions of 160 × 40 × 40 mm (Figure 3) were created by filling the plastic molds with a plaster mixture [50]. After the mold was filled, air was expelled from the samples, and the surfaces of the samples were smoothed. Due to the sizes of the samples and the presence of PCM, the samples still had high humidity after 28 days. The humidity of the samples for strength testing was measured by the gravimetric method, and the humidity of the samples applied to the aerated concrete blocks was determined with capacitive moisture measurement device GREISINGER GMK 100. While reference samples S0 and R0 had a humidity of about 5% and could be tested, samples with PCM had a humidity of 15% to 20%. PCM slowed the drying and setting of these larger samples. Therefore, the samples were stored in the test environment (temperature 23 ± 2 °C and relative humidity of the air 50 ± 5%) for three months. Before testing, all samples were conditioned at 40 °C for 48 h, then left in the test environment for another 2 h.

### 2.3. Test Methods

All samples were weighed and the dimensions of the samples removed from the molds were measured. The volume of these samples was calculated and their bulk density *ρ* (kg·m^−3^) was determined. All tests were carried out according to valid standards. The indoor air temperature and relative humidity during sample testing was 24 °C and 45.0%, respectively.

#### 2.3.1. Flexural Strength

**Figure 4:**
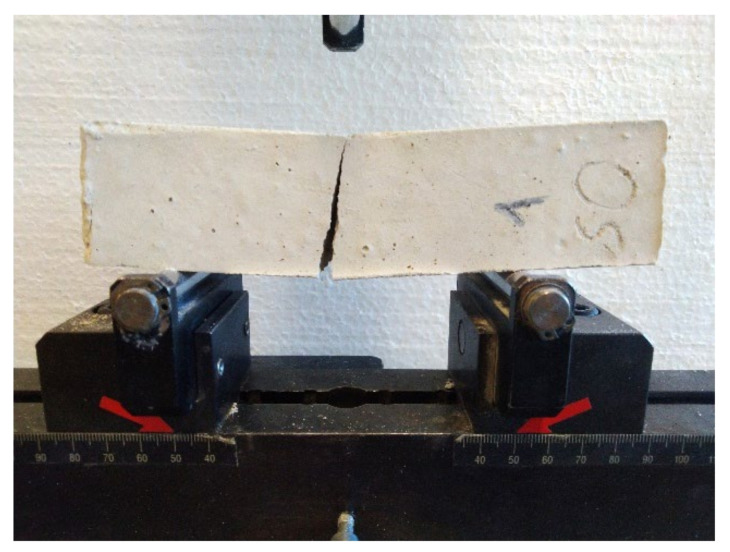
Sample after the flexural strength test.

The samples were placed sideways in the FormTest press on supports spaced 100 mm apart. The longitudinal axis of the samples was perpendicular to the supports of the press. The load was transmitted through the load roller perpendicular to the sample surface. The load was evenly increased at 10 N/s until the sample broke (Figure 4). The force that caused the break of the sample was recorded. The flexural strength *P_F_* (MPa) was calculated according to Equation (1).

(1) $$P_{F}=0.00234\cdot P$$

where *P* (N) is the maximum applied load [50].

#### 2.3.2. Compressive Strength

**Figure 5:**
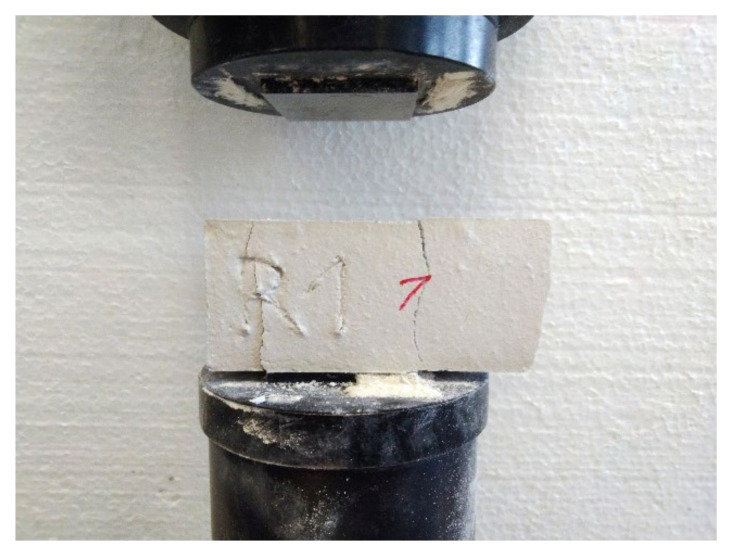
Sample after the compressive strength test.

The halves of the test samples broken in the flexural strength test were tested for compressive strength over an area of 40 × 40 mm. The samples were placed sideways on the FormTest press and centered relative to the press plates. The load was evenly increased at 50 N/s until the failure occurred in the samples (Figure 5) and the peak load was reached. The maximum force was recorded. The compressive strength *R_C_* (MPa) was calculated according to Equation (2).

(2) $$R_{C}=\frac{F_{C}}{1600}$$

where *F_c_* (N) is the maximum applied load [50].

#### 2.3.3. Tensile Strength Perpendicular to the Surface

**Figure 6:**
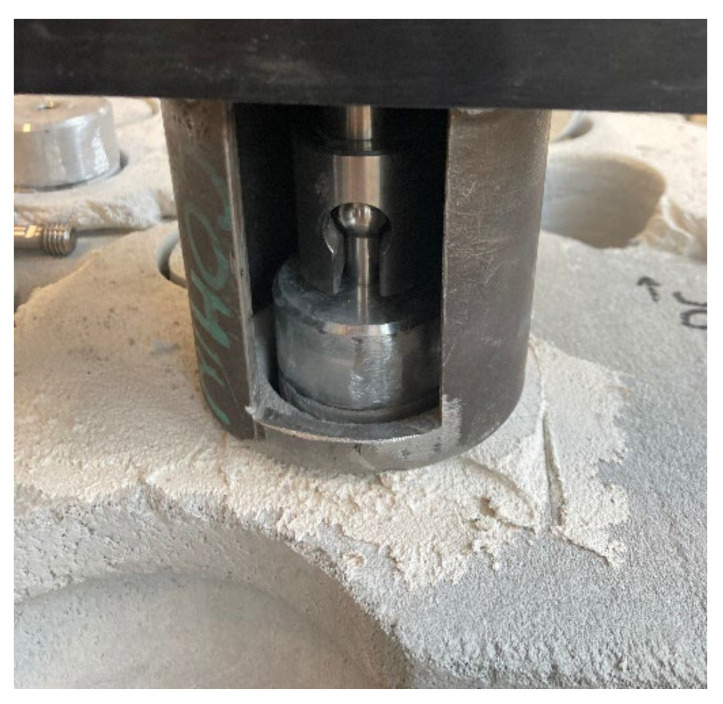
Measurement of the tensile strength perpendicular to the surface.

First, test cylinders with a diameter of 50 mm were drilled into the plaster layer. Drilling was carried out to a depth of 5 mm in the aerated concrete blocks. Circular steel targets were centrically glued to the surfaces of the cylinders with cyanoacrylate glue. The tensile load was applied perpendicular to the test surface over the circular targets using a Comtest OP3 test device (Figure 6). The load was evenly increased at 5 N/s until the sample was torn off. The maximum applied load and the way the sample was torn off were recorded. The tensile strength perpendicular to the surface *R_u_* (MPa) was calculated according to Equation (3).

(3) $$R_{u}=\frac{F_{u}}{A}$$

where *F_u_* (N) is the maximum applied load and *A* (mm^2^) is the test area of the cylindrical sample [50].

#### 2.3.4. Thermal Conductivity

**Figure 7:**
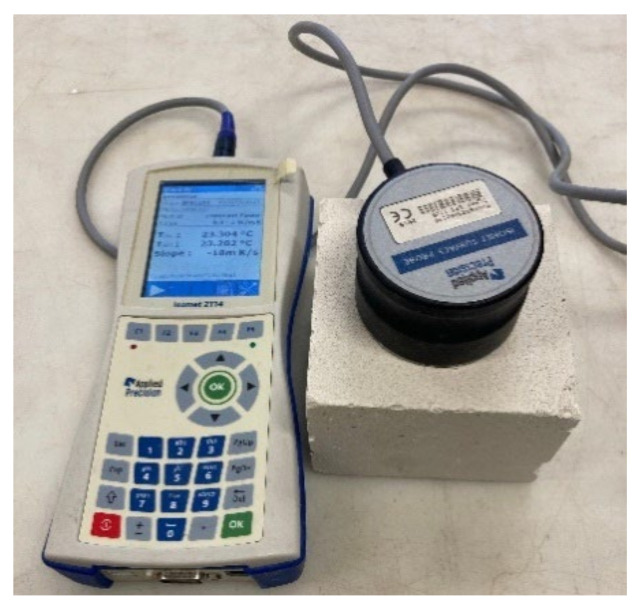
ISOMET device with the surface probe on the sample.

The thermal conductivity coefficient was measured with the ISOMET 2114 device equipped with a surface probe (Figure 7). The surface probe was placed sequentially on the surface of each sample. This device uses a non-stationary hot wire method for measurement [52]. The principle of this method is to record temperature rises and falls at a defined distance from the heat source, that is, the hot wire. The ISOMET 2114 device records the power per unit length, temperatures, and times and calculates the thermal conductivity coefficient *λ* (W·m^−1^·K^−1^) [53].

#### 2.3.5. Specific Heat Capacity

The specific heat capacity was also determined using an ISOMET 2114 device with a surface probe. The measuring principle is the same as that of the thermal conductivity measurements [52]. The device records the amount of heat per unit volume and changes in temperature and calculates the volumetric heat capacity *C_ρ_* (J·m^−3^·K^−1^) [53]. Based on the bulk density of individual samples, the specific heat capacity *c* (J·kg^−1^·K^−1^) was calculated according to Equation (4).

(4) $$c=\frac{C_{\rho }}{\rho }$$

where *C_ρ_* (J·m^−3^·K^−1^) is the volumetric heat capacity and *ρ* (kg·m^−3^) is the bulk density.

#### 2.3.6. Differential Scanning Calorimetry

DSC analysis was performed using a 3D DSC calorimeter Setaram SENSYS EVO in horizontal mode equipped with a unique 3D DSC sensor [54]. Absolute enthalpy calibration by use of Joule effect was performed. Temperature calibration was performed using high purity of In (5N). All the samples were cyclic analyzed. Heating and cooling followed by second heating and cooling were performed at the rate of 2 °C/min in He (6N) atmosphere. The powdery samples were analyzed in corundum “boats”. The masses of samples were between 50 and 147 mg.

**Figure 8:**
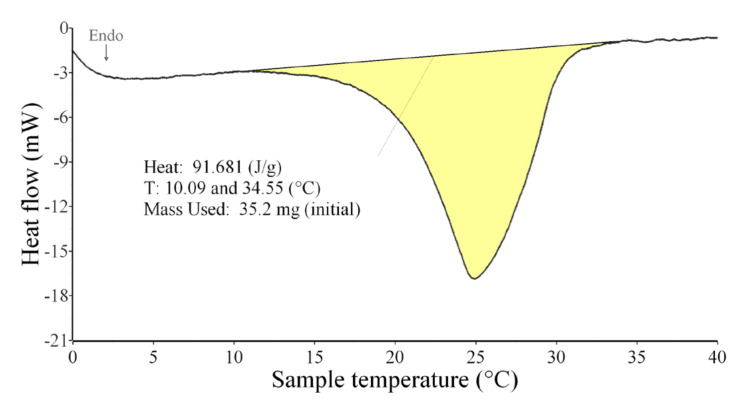
DSC curve of pure PCM, peak start and end temperature, and area of the peak (heat absorbed)—heating.

**Figure 9:**
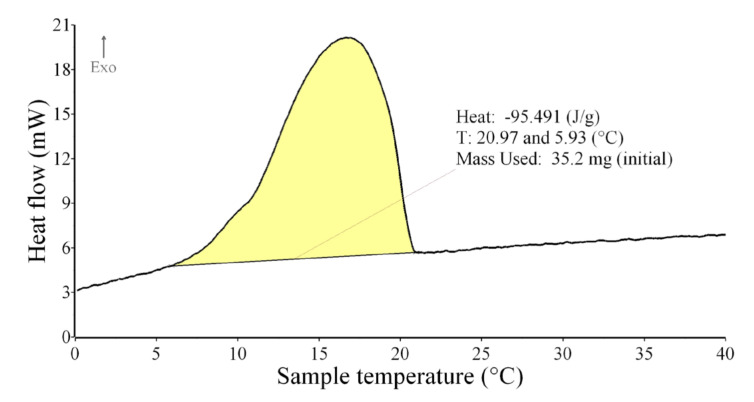
DSC curve of pure PCM, peak start and end temperature, area of the peak (heat released)—cooling.

The enthalpy (heat effects) of melting and solidification of samples—PCM in prepared samples—were measured. The enthalpy is demonstrated by the peak areas (yellow areas) in Figure 8 and Figure 9. The first observed deviation (at 10 °C) from the base line was taken as the start of the melting and the end of the peak (35 °C) denotes the end of the melting process as presented on the DSC curve of pure PCM (Figure 8). The same procedure for peak area evaluation was used for the cooling process. The first observed deviation (at 21 °C) from the base line was taken as the start of the solidification and the end of the peak (6 °C) denotes the end of the solidification process as presented on the DSC curve of the pure PCM (Figure 9). Mean values of enthalpy of melting (from two heating runs) and solidification (from two cooling runs) were calculated. The mean values of start and end of melting and solidification were obtained.

#### 2.3.7. Single-Flame Source Fire Test

**Figure 10:**
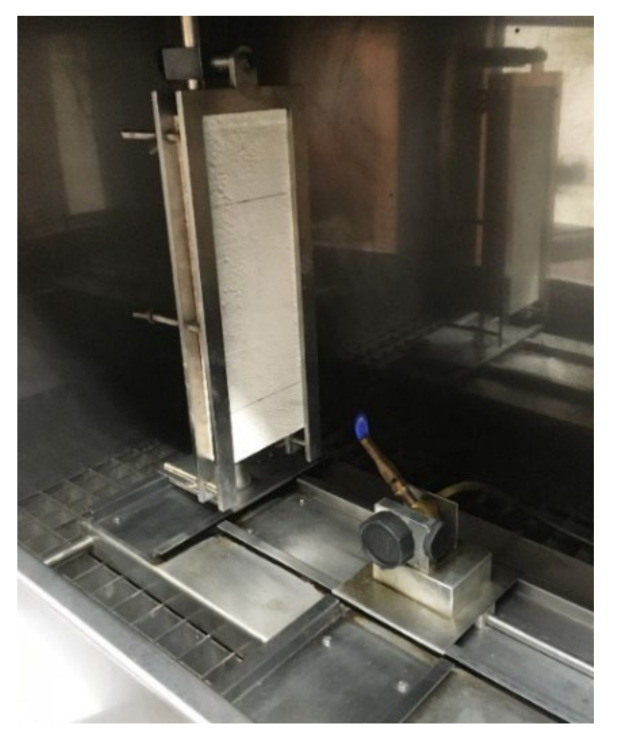
Sample in the combustion chamber.

The ignitability of samples subjected to direct impingement of flame was measured by a single-flame source test in a combustion chamber. Two horizontal axes were marked on the exposed surfaces of the samples. The first axis was 40 mm from the bottom edge of the sample. The second axis was at a distance of 150 mm from the first axis. The first axis determined where the flame of the gas burner touched the surface of the measured sample. The flame height was set at 20 mm, then the gas burner was tilted 45° with respect to the vertical axis (Figure 10). The samples were exposed to flame for 30 s. During the test, it was recorded whether ignition occurred and whether the flame front exceeded the second axis, as well as the time at which this occurred. The physical behavior of the test samples was observed during the test [51]. First, the samples with the highest PCM proportion (S6, S7, and R4) were measured, and if these samples passed this test, the samples with the lower PCM proportion would also pass.

#### 2.3.8. Gross Heat of Combustion

**Figure 11:**
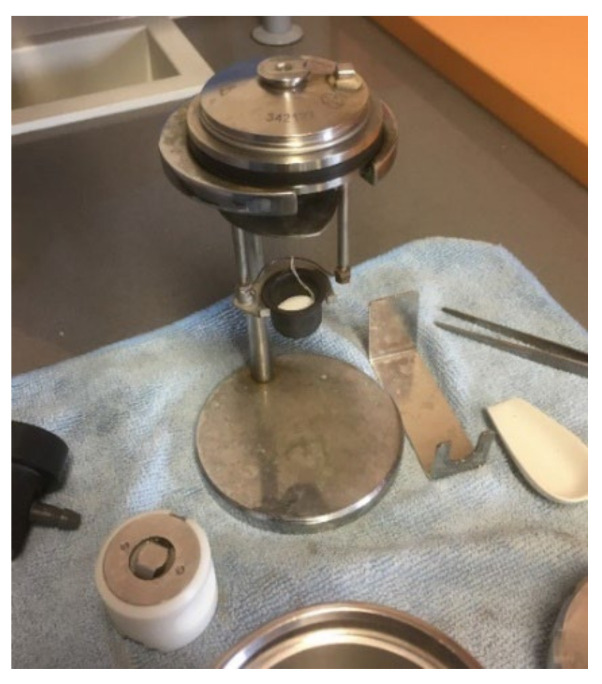
Sample in the crucible of the bomb calorimeter.

The gross heat of combustion (calorific value) *Q_PCS_* (MJ·kg^−1^) was measured in an oxygen bomb calorimeter IKA C 200. The measurement was performed under standardized isoperibolic conditions, at constant volume, and in an oxygen atmosphere. The test sample was burned in a bomb calorimeter using the crucible method, and the gross heat of combustion was calculated on the basis of the observed temperature rise, taking into account the heat loss and the latent heat of water vaporization [55]. As gypsum and sand are non-flammable, only the gross heat of combustion of the pure PCM was measured (Figure 11). PCM is a fine powder; the sample was compressed with an IKA pelleting press prior to measurement. The mass of the samples was approximately 0.50 g. The gross heat of combustion of the other samples was calculated from the measured value according to the percentage of PCM in the mixture.

## 3. Results and Discussion

Each test was carried out according to standards for several samples. The average of the measured values was calculated. The average values of the quantities are given in Table 3, Table 4, Table 5 and Table 6.

### 3.1. Mechanical Properties

Table 3 and Table 4 show the measured values of the physical and mechanical properties of all samples from set S and set R. The values of the bulk density *ρ*, flexural strength *P_F_*, compressive strength *R_C_*, and tensile strength perpendicular to the surface *R_u_* are given in the Tables.

**Figure 12:**
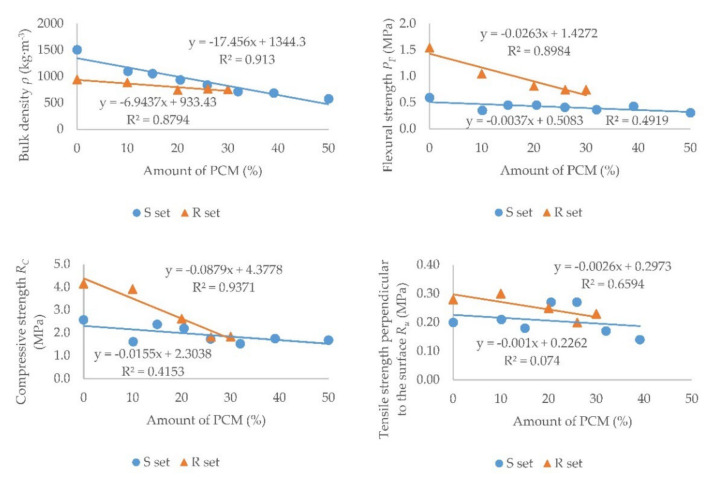
Dependence of mechanical properties on the amount of PCM.

In Figure 12, the measured values of the quantities depending on the amount of PCM in the sample are shown in the graphs. Set S is marked in blue and set R is marked in orange. A linear trend line was created and its equation was determined. The value for sample S7 was excluded from the tensile strength perpendicular to the surface graph. This sample was special because it did not contain sand, which has a larger particle diameter than PCM. The sand had a particle size of 0.09 to 1.25 mm and the PCM had a particle size of 5 to 20 μm [27], which probably caused a lower porosity and thus a higher measured value.

It can be seen that the bulk density decreased with an increasing proportion of PCM in the sample. This decrease was greater in the set S, as the lightweight PCM replaced the sand with a higher bulk density. The decrease was smaller in the R set, because Rigips Rimano UNI plaster contains a lightweight expanded perlite as the aggregate [49]. The addition of PCM reduced the proportion of the gypsum binder in the mixture, causing a slight decrease in bulk density.

As expected, all strengths were reduced, as has been proven, e.g., in [8,9]. It can be seen in the graphs that the decrease in set S was smaller than that in set R. This is because in set S the ratio of the binder to the aggregate was still the same, because part of the sand was replaced by PCM. The strength of set R decreased significantly with an increasing proportion of PCM as a result of the increase in the proportion of the aggregate to the binder. It can also be seen that the flexural and compressive strength of set R was higher than that of set S. This was due to the additives in the commercial plaster mixture Rigips Rimano UNI. On the contrary, no additives were added to set S. Sample S1 had lower flexural and compressive strength values, which could be due to material inhomogeneity caused by the mold filling. It is clear from the tensile strength perpendicular to the surface graph that the trend was generally decreasing, but a few values did not follow the trend. Samples S3 and S4 had a higher value of the tensile strength perpendicular to the surface than might be expected. This could be caused, for example, by the inhomogeneity of the material. When measuring the tensile strength perpendicular to the surface, all samples were torn off in a plaster layer. No sample was torn off at the contact of the plaster with the aerated concrete block.

The standard [17] specifies the requirements for the strength of gypsum binders and mortars for internal plasters. The minimum required flexural strength is 1 MPa, which was met only by samples R0 and R1. The minimum required compressive strength is 2 MPa, which was met by samples S0, S2, S3, R0, R1, and R2. When measuring the tensile strength perpendicular to the surface, the standard [17] requires the sample to be torn off in the plaster layer, which all samples met. Set S without any additives was considered as a reference for plasters in which the aggregate is replaced by PCM, and set R was a reference for plasters in which only PCM is only added. To increase the strength of set S, it is possible to use another type of gypsum and additives [48,56,57] that are commonly added to plaster mixtures. Polyvinyl acetate, furfural, fumed silica, carbon fibers, or polypropylene fibers could be used to increase the flexural strength [58,59,60]. On the other hand, the results of the R set are difficult to improve, because the samples already contain the additive. For set R, the requirements of the standard [17] can be met with a maximum of 10% PCM in the mixture. According to [6], it is possible to meet the requirements of the standard [17] for gypsum plasters with the replacement of the aggregates with PCM at a maximum amount of 30% PCM in the mixture. When replacing part of the aggregate with PCM in the commercial gypsum plaster mixture Rigips Rimano UNI, higher strength values can be obtained while following the trend according to set S, which can meet the requirements of the standard [17] even at 30% PCM. This needs to be confirmed by further research into plasters with different additives.

### 3.2. Thermal Properties

Table 5 and Table 6 show the measured values of the thermal properties of all samples of set S, set R, and PCM. The values of the thermal conductivity coefficient *λ*, specific heat capacity *c*, temperatures at the start and end of melting/solidification, and enthalpy of the melting and solidification are given in the Tables.

**Figure 13:**
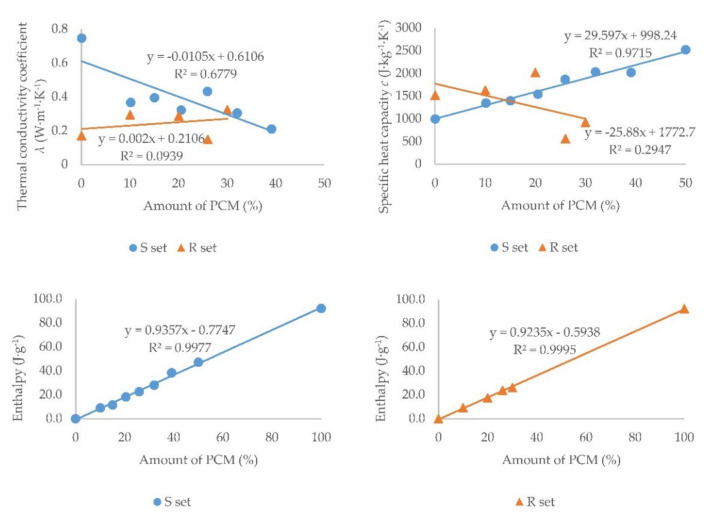
Dependence of thermal properties on the amount of PCM.

Figure 13 presents the measured values of the quantities depending on the amount of PCM in the sample. Set S is marked in blue and set R is marked in orange. A linear trend line was created and its equation was determined. The value for sample S7 was excluded from the thermal conductivity coefficient graph. The absence of sand in the sample probably caused a lower porosity in the sample because PCM has a smaller particle diameter than the sand. The sand had a particle size of 0.09 to 1.25 mm and PCM has a particle size of 5 to 20 μm [27]. This might have also caused a higher value of the thermal conductivity coefficient. The enthalpy values of melting were plotted regarding the PCM content (Figure 13). The dependence of the enthalpy of solidification on the PCM content was almost the same.

As expected, with an increasing proportion of PCM, a decrease in the thermal conductivity coefficient and an increase in the specific heat capacity was observed for set S. This is because PCM has a lower thermal conductivity and a higher specific heat capacity compared to sand. In set R, some values did not follow the trend, which is also evidenced by the trendline’s reliability; however, in general, it can be said that with an increasing proportion of PCM, the thermal conductivity coefficient increased slightly and the specific heat capacity decreased. All measured values of the thermal conductivity coefficient of the set R were very low. But the value of the thermal conductivity of sample R3 was very low compared to the other samples. For all R3 samples, the value of the thermal conductivity coefficient ranged from 0.14 to 0.16 W·m^−1^·K^−1^. The expanded perlite in the commercial plaster mixture Rigips Rimano UNI had a lower thermal conductivity and a higher specific heat capacity compared to PCM. When using a commercial plaster mixture with a higher bulk density, it can be expected that as the proportion of PCM in the mixture increases, the thermal conductivity coefficient will decrease and the specific heat capacity will increase, as has been proven, e.g., in [24,25,26]. When building structures are designed, a low thermal conductivity coefficient and thus better thermal insulation properties of the structure are usually desirable. However, in the case of plasters with PCM, higher values of the thermal conductivity coefficient are more suitable, because they shorten the response times of plasters to increases or decreases in indoor air temperatures in summer. However, slightly lower values of the thermal conductivity coefficient, and thus slightly longer response times of plasters to indoor air temperatures, do not prevent their use in interiors. Hypothetically, a small amount of natural or expanded graphite [22] or aluminum [36] could be added to the mixture to increase the thermal conductivity coefficient.

Since the phase change in a plaster mixture occurs only in the PCM, the amount of heat stored/released should therefore be proportional to the percentage of PCM in the plaster. This assumption was confirmed by results obtained, see Table 5 and Figure 13. The measured enthalpy of melting of pure PCM (100% PCM) was 92.1 J·g^−1^ and the enthalpy of solidifying was 97.3 J·g^−1^, the highest measured values. The DSC analysis of the other samples (plaster mixtures with graded PCM content) confirmed that the enthalpy values vary correspondingly with the percentage of PCM contained in the mixture (the higher the PCM content, the higher the heat absorbed or released—linear dependence in S and R sets was obtained). The heat absorption took place in a wider temperature interval and at higher temperatures compared to the cooling process, in which the heat was released in a narrower temperature interval and at lower temperatures; see Figure 8 and Figure 9 and the temperature intervals in Table 5 and Table 6.

In order to know how PCM actually affects the course of indoor air temperatures, a comparative measurement was performed in 2019 in identical attic rooms in Brno in the Czech Republic. In the first room there was plaster without PCM and in the second there was plaster with 30% PCM. It was found that gypsum plaster with 30% PCM could reduce the maximum daily temperature by up to 2.6 °C [61].

### 3.3. Fire Properties

**Figure 14:**
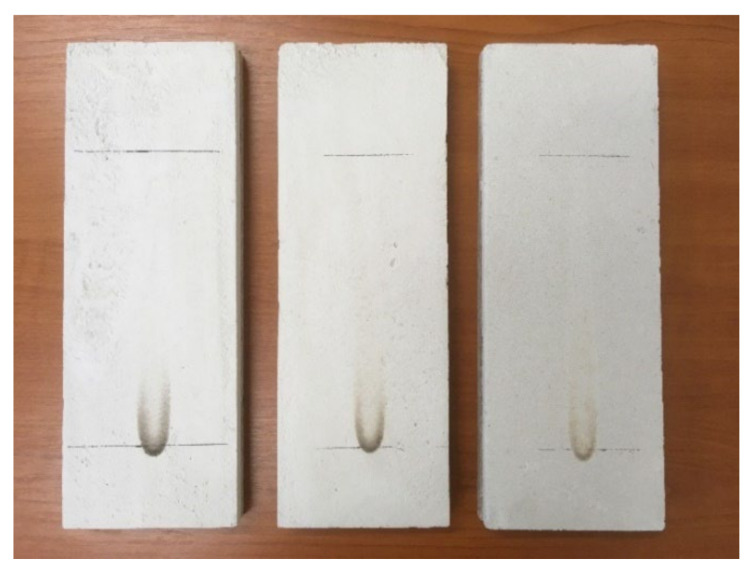
Samples after the single-flame source fire test.

Figure 14 shows the test samples after the single-flame source fire test. Only the samples with the highest PCM proportion were measured. Figure 14 shows samples S7, S6, and R4 from the left. It can be seen that even after 30 s, the flame did not reach the second axis. This means that these samples can be classified as better than reaction to fire class E [47]. Reaction to fire classes E and F [47] could also be excluded for other samples with lower PCM proportions.

During the single-flame source fire test, the surfaces of the test samples did not ignite and the fire did not spread. There was no release of smoke or odor. Reaction to fire classes C and D [47] exclusions can also be expected based on the behavior of the samples during this fire test. A gross heat of combustion measurement was performed to classify the samples into classes A1, A2, and B [47]. As gypsum and aggregates are non-flammable, the proportion of flammable PCM in the mixture is crucial for determining the reaction to fire class. The gross heat of combustion was determined on the pure PCM Micronal DS 5008 X and calculated according to the percentage of PCM in each sample. The gross heat of combustion (calorific value) *Q_PCS_* of PCM was 37.81 MJ·kg^−1^. To classify a sample into class A, the gross heat of combustion must be less than 3 MJ·kg^−1^ [47]. This means that all samples, except reference samples with 0% PCM, would be classified into reaction to fire class B [47].

The standard [18] requires that interior plasters attain at least a reaction to fire class B. In the case of plasters on protected escape routes, only plasters with reaction to fire classes A1 or A2 may be used. Plasters with PCM are expected to be used in habitable rooms, where class B is sufficient. All tested plasters meet the requirement of the standard [18] for this reaction to fire class.

## 4. Conclusions

Investigating the properties of plasters with PCM is very important for their future use. The composition of plasters can vary by binder, aggregate, additives, and type of PCM. This research focused on the investigation of two different approaches to the integration of microencapsulated PCM into gypsum plaster. The first approach was to replace part of the aggregate by PCM (set S); in the second approach, PCM was only added to the commercial dry plaster mixture (set R). The aim was to study the dependence of the basic properties of plasters on the amount of PCM contained in the plaster mixture in both approaches. The main findings are as follows:

- It was observed that in samples of both sets, with an increasing proportion of PCM, the bulk density, flexural strength, compressive strength, and tensile strength perpendicular to the surface decreased.
- When comparing the trends of the S and R sets for their mechanical properties, it can be stated that in set R there was a more significant decrease in these strengths than in set S.
- With an increasing proportion of PCM in samples, the enthalpy increased. In set S, the thermal conductivity coefficient decreased and the specific heat capacity increased. In set R, the thermal conductivity coefficient slightly increased and the specific heat capacity decreased due to the low thermal conductivity of the reference sample R0 (0.171 W·m^−1^·K^−1^) caused by the expanded perlite.
- DSC analysis showed that the enthalpy was proportional to the amount of PCM contained in the plaster. Lower values of the thermal conductivity coefficient for PCM plasters are not ideal, but do not prevent their use in interiors.
- For samples with PCM, the fire properties of the plasters deteriorated, and all were classified into reaction to fire class B due to the high value of the gross heat of combustion. Class B does not prevent the use of plaster in habitable rooms, but it does not allow their use on protected escape routes.
- In terms of standard requirements, only plasters up to 10% PCM can be used in the manner of set R for common commercial plaster mixtures. However, the advantage of the R set is its easy production directly on the construction site. In the manner of set S, according to the determined trends, the applicability of plasters up to approximately 30% PCM can be hypothetically assumed after the addition of additives.

## Figures and Tables

**Table 1:** Mixture composition of the S set.

Sample	Gypsum (g)	Water (g)	Sand (g)	PCM (g)	PCM (%)
S0	500	700	2000	0	0
S1	500	700	1200	190	10.1
S2	500	700	950	255	15.0
S3	500	700	660	300	20.5
S4	500	700	500	350	25.9
S5	500	700	350	400	32.0
S6	500	700	200	450	39.1
S7	500	700	0	500	50.0

**Table 2:** Mixture composition of the R set.

Sample	Gypsum Plaster (g)	Water (g)	PCM (g)	PCM (%)
R0	1000	600	0	0
R1	900	540	100	10
R2	800	480	200	20
R3	700	420	250	26
R4	700	420	300	30

**Table 3:** Mechanical properties of the S set.

Sample	Bulk Density *ρ* (kg·m^−3^)		Flexural Strength *P_F_* (MPa)		Compressive Strength *R_C_* (MPa)		Tensile Strength Perpendicular to the Surface *R_u_* (MPa)	
	Average	Standard Deviation	Average	Standard Deviation	Average	Standard Deviation	Average	Standard Deviation
S0	1502	22	0.59	0.02	2.57	0.12	0.20	0.03
S1	1096	5	0.35	0.01	1.61	0.08	0.21	0.01
S2	1053	4	0.45	0.03	2.37	0.10	0.18	0.03
S3	933	9	0.45	0.07	2.20	0.08	0.27	0.04
S4	836	7	0.41	0.02	1.74	0.10	0.27	0.03
S5	714	8	0.37	0.04	1.52	0.10	0.17	0.02
S6	683	10	0.43	0.04	1.75	0.07	0.14	0.01
S7	575	7	0.30	0.05	1.68	0.09	0.41	0.04

**Table 4:** Mechanical properties of the R set.

Sample	Bulk Density *ρ* (kg·m^−3^)		Flexural Strength *P_F_* (MPa)		Compressive Strength *R_C_* (MPa)		Tensile Strength Perpendicular to the Surface *R_u_* (MPa)	
	Average	Standard Deviation	Average	Standard Deviation	Average	Standard Deviation	Average	Standard Deviation
R0	940	21	1.54	0.03	4.14	0.33	0.28	0.03
R1	880	3	1.04	0.05	3.91	0.27	0.30	0.04
R2	740	2	0.81	0.03	2.62	0.14	0.25	0.01
R3	760	4	0.74	0.03	1.82	0.06	0.20	0.01
R4	750	9	0.74	0.04	1.84	0.07	0.23	0.04

**Table 5:** Thermal properties of the S set and PCM.

Sample	Thermal Conductivity Coefficient *λ* (W·m^−1^·K^−1^)		Specific Heat Capacity *c* (J·kg^−1^·K^−1^)		Temperatures at Start and End (°C)		Enthalpy (J·g^−1^)	
	Average	Standard Deviation	Average	Standard Deviation	Melting	Solidification	Melting	Solidification
S0	0.747	0.016	989.95	4.63	–	–	0	0
S1	0.367	0.011	1340.79	3.79	12–31	22–7	8.9	−10.1
S2	0.394	0.033	1391.07	22.13	12–30	22–7	11.5	−12.9
S3	0.321	0.019	1540.62	13.24	12–31	21–7	18.2	−19.9
S4	0.431	0.018	1864.12	8.19	12–31	22–6	22.6	−24.9
S5	0.304	0.009	2029.41	7.33	10–32	21–5	28.0	−31.2
S6	0.210	0.013	2013.91	6.67	9–32	21–5	38.3	−41.9
S7	0.533	0.009	2516.52	26.78	9–33	21–5	47.2	−50.3
PCM	–		–		9–34	21–5	92.1	−97.3

**Table 6:** Thermal properties of the R set.

Sample	Thermal Conductivity Coefficient *λ* (W·m^−1^·K^−1^)		Specific Heat Capacity *c* (J·kg^−1^·K^−1^)		Temperatures at Start and End (°C)		Enthalpy (J·g^−1^)	
	Average	Standard Deviation	Average	Standard Deviation	Melting	Solidification	Melting	Solidification
R0	0.171	0.022	1515.85	15.20	–	–	0	0
R1	0.294	0.012	1626.36	6.64	11–30	22–9	9.2	−10.1
R2	0.287	0.015	2016.76	18.82	12–31	22–7	17.3	−19.6
R3	0.149	0.011	559.61	4.21	11–31	21–8	23.7	−25.1
R4	0.322	0.009	919.20	21.42	11–32	21–7	25.9	−27.9
